# Platelet Rich Plasma Enhancement of Skin Regeneration in an *ex-vivo* Human Experimental Model

**DOI:** 10.3389/fbioe.2019.00002

**Published:** 2019-01-22

**Authors:** Giovanni Nicoletti, Marco Saler, Laura Villani, Agnese Rumolo, Marco Mario Tresoldi, Angela Faga

**Affiliations:** ^1^Plastic and Reconstructive Surgery, Department of Clinical Surgical, Diagnostic and Pediatric Sciences, University of Pavia, Pavia, Italy; ^2^Advanced Technologies for Regenerative Medicine and Inductive Surgery Research Center, University of Pavia, Pavia, Italy; ^3^Plastic and Reconstructive Surgery Unit, Department of Surgery, Istituti Clinici Scientifici Maugeri, Pavia, Italy; ^4^Pathological Anatomy and Histology Unit, Istituti Clinici Scientifici Maugeri, Pavia, Italy; ^5^Department of Molecular Medicine, University of Pavia, Pavia, Italy

**Keywords:** platelet rich plasma, regeneration, culture techniques, *ex-vivo*, skin, collagen, elastic fibers

## Abstract

This study reports on the development of an original, *ex-vivo* wounded skin culture protocol using autologous Platelet Rich Plasma (PRP) and enriched Dulbecco's Modified Eagle's Medium (DMEM). Human skin samples obtained from specimens harvested during reduction mammoplasty procedures, were injured in their central portion—to create a standard wound—and cultured under three different conditions:

– enriched DMEM with saline solution in the central wound (control)

– enriched DMEM with the same medium in the central wound

– enriched DMEM plus 2.5% autologous PRP, with the same PRP added medium in the central wound.

Morphological analysis was carried out at 0 h (T_0_) and on days 1, 3, 5 and 10 (T_1_-T_3_-T_5_-T_10_) using Hematoxylin and Eosin; Masson's trichrome staining; Weigert staining and Ki-67 staining to identify the skin histological features in the different experimental conditions. The combination of DMEM and PRP allowed a favorable modulation of the epithelial cells and fibroblasts proliferation, and a relevant anti-inflammatory action. PRP also demonstrated an inhibitory effect on both the collagen and elastic fibers' de-structuration and a favorable modulation of the re-organization of these fibers. The step by step histological and immune-histo-chemical regenerative effects of PRP on human skin wound repair and regeneration process was observed over a period of 10 days.

## Introduction

Several experimental models are currently available for the investigation of complex skin physiology.

The *in-vitro* models are usually made of a single skin cell type—fibroblasts or keratinocytes—cultured in the appropriate medium (Johnen et al., 2008). The main advantages of the *in-vitro* models are their low cost and ease of set-up. The most relevant disadvantage is the impossibility of reproducing the three-dimensional structure and complex physiology of whole skin. The *in-vivo* models are both animal and human. The animal *in-vivo* models allow a huge variety of experimental options. The disadvantages are the fine differences in skin physiology between humans and animals and the ethical issues that strongly limit this model. Human *in-vivo* models are strongly discouraged for reasons of both ethics and high costs. The *in-silico* skin model is considered the main computational one, and can complement other models in investigating skin behavior (Ud-Din and Bayat, 2017). The *in-virtuo* model was developed by gathering all of the currently available data on skin cellular interactions, vitality and gene expression. In spite of this, there remains a significant lack of essential data which would support the consideration of this model as a close approximation to normal skin (Lebonvallet et al., 2010). The *ex-vivo* organotypic model (3D) allows the best approximation to living, human skin. The model may be bioengineered or native. Although the bioengineered *ex-vivo* organotypic model allows for control of the keratinocytes' differentiation, it does not include all of the different cell types of living skin. It lacks, for example, the skin adnexa and, its manufacturing process is time and money consuming. In fact, the model which best approximates living human skin, is the organotypic model as it enables immediate and short-term evaluation of a particular effect on cells and surrounding tissue components, although it is seldom used due to its technical complexity and the limited availability of full thickness human skin samples (Safferling et al., 2013; Mori et al., 2016).

Among the different wound healing culture models previously reported (Tomic-Canic et al., 2007; Peramo et al., 2010; Xu et al., 2012), the *ex-vivo* donut-shaped model has been largely used to investigate human cutaneous repair in several experiments (Hodgkinson and Bayat, 2016; Ud-Din and Bayat, 2017).

The aim of this study is the development of an original, *ex-vivo* human wounded skin culture protocol by adding autologous Platelet Rich Plasma (PRP) to a conventional culture medium, in order to enhance the tissue regeneration process. PRP is one of the most versatile tools in Regenerative Medicine as it is easily extracted from peripheral blood samples and allows a platelet count 3 to 5 folds higher than normal, thus providing a huge variety of highly concentrated active Growth Factors.

## Materials and Methods

The project was conducted in collaboration between the Plastic and Reconstructive Surgery Unit, the Pathological Anatomy Section Laboratory of the ICS Maugeri SB SpA IRCCS in Pavia (Italy), and the Immunology and General Pathology Laboratory of the Department of Molecular Medicine of the University of Pavia (Italy). The study conformed to the 1975 Declaration of Helsinki: an informed written consent was obtained from all of the patients and the protocol was approved by the Ethics Committee of the ICS Maugeri SB SpA IRCCS, Pavia (Italy) (project identification code, 2064).

### Human Skin Specimen Collection

Human skin samples were obtained from anatomical specimens harvested during sessions of reduction mammoplasty, performed on 10 healthy female patients with an age range of 43–60 years. The specimens were sampled by a surgeon in 8 x 8 cm fragments, stored in sterile containers filled with sterile saline solution (S.A.L.F. SpA, Cenate Sotto, Bergamo, Italy) enriched with 1% (10,000 U/ml) penicillin and streptomycin (10 mg/ml) (Sigma-Aldrich; Merck KGaA, Darmstadt, Germany) and then transported in ice to the Immunology and General Pathology Laboratory for further processing. The time lag between tissue harvesting and the start of the laboratory procedures was approximately 15 min.

### PRP Preparation

The Tissue Regeneration Kit (New Technologies Supplies, Latina, Italy) was used for preparation of PRP according to the Okuda protocol (Okuda et al., 2003). The kit was equipped with a vacutainer tool and a 12 ml tube provided with sodium citrate anticoagulant and a magnetic polymer separator gel. One hour before the surgical procedure, 10 ml of blood were aspirated from the patient's peripheral vein in the dedicated tube. The tube was gently shaken 5 times and then centrifuged at 1500 G for 5 min, thus obtaining 4 phases: platelet-poor plasma, buffy coat (enriched platelets), separator gel and blood cells. The tube was then gently inverted for 20 s to re-suspend the platelets. Eventually, ~4 ml of pure platelet suspension (PRP)—without fibrinogen, leukocytes or red blood cells—was aspirated with a syringe, and, kept on ice, transported to the laboratory.

### Human Skin Specimen Processing

**Figure 1:**
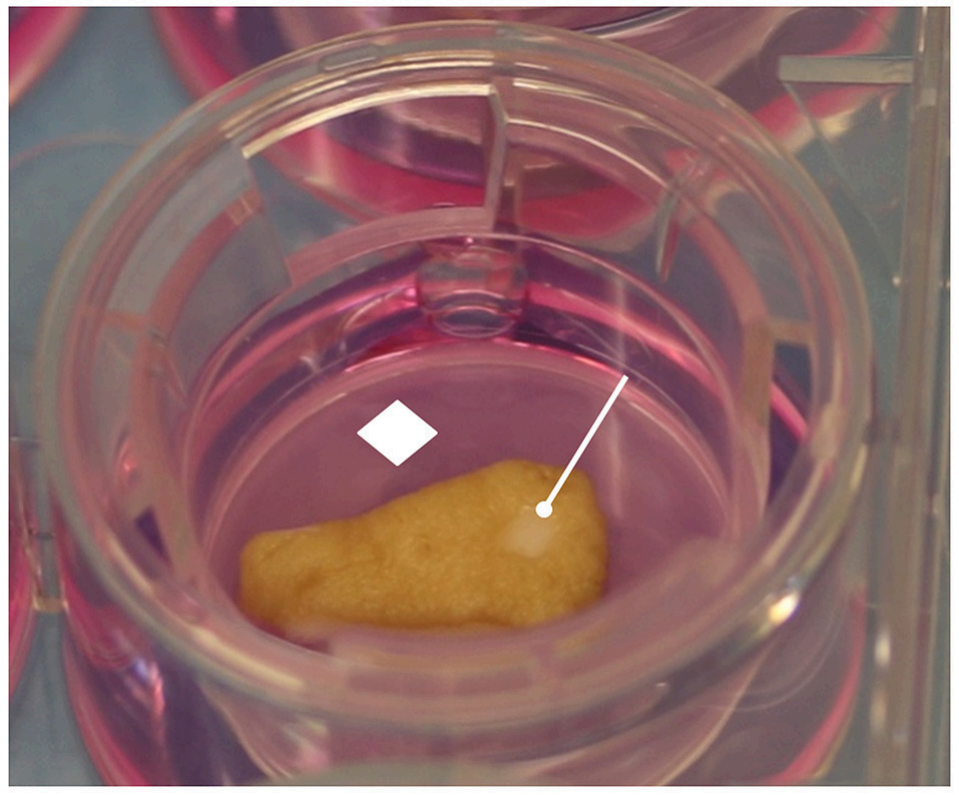
Human skin specimen processing. The skin samples were cultured in Transwell inserts, in contact with culture medium by porous membrane. The epidermis and wound area were exposed to the air. White rhombus = porous membrane; white drumsticks = standard central injury.

Human skin specimens were processed in a sterile environment provided with a second-class laminar flow hood. Each specimen was divided into multiple 1 cm^2^ full thickness punch biopsies that in turn were injured in their central portion with a sterile 3 mm circular punch (Blife, Casale sul Sile, Treviso, Italy), in order to create a standard split thickness skin loss involving the epidermis and the superficial dermis (Mori et al., 2016; Nicoletti et al., 2017). Each injured sample was placed in Transwell inserts (6-well multiwell plates, membrane pore size, 0.40 μm; Constar insert, 0.33 cm^2^; Corning Incorporated, Corning, NY, USA) inside the wells. The medium added into the well absorbed the samples through a porous membrane, while the superficial layers of the dermis and the epithelium were exposed to the air (Figure 1).

### Study Design

The skin specimens, harvested from 10 different patients, allowed for the preparation of paired control and experimental samples for each donor. The control samples (C) were cultured in Dulbecco's Modified Eagle's Medium powder with 4,500 mg/l glucose, 0.584 g/l L-glutamine and 0.11 g/l sodium pyruvate (DMEM) (Sigma-Aldrich), reconstituted with distilled water (Milli-Q, Merck-Millipore, Darmastadt, Germany) and enriched with 3.7 g/l sodium bicarbonate, 10% fetal bovine serum (FBS), 1% (10,000 U/ml) penicillin and streptomycin (10 mg/ml) (all from Sigma-Aldrich); the central skin loss region was treated with a constant volume (20 μl) of sterile saline solution (S.A.L.F. SpA). The experimental samples were cultured in two different conditions:

– in the first experimental condition (D) the samples were cultured with DMEM reconstituted with distilled water (Milli-Q, Merck-Millipore, Darmastadt, Germany) and enriched with 3.7 g/l sodium bicarbonate, 10% fetal bovine serum (FBS), 1% (10,000 U/ml) penicillin and streptomycin (10 mg/ml) (all from Sigma-Aldrich); a constant volume (20 μl) of the same culture medium was applied in the central skin loss region, too.
– in the second experimental condition (DP) the samples were cultured with DMEM reconstituted with distilled water (Milli-Q, Merck-Millipore, Darmastadt, Germany), enriched with 3.7 g/l sodium bicarbonate, 10% FBS, 1% (10000 U/ml) penicillin and streptomycin (10 mg/ml) (all from Sigma-Aldrich) plus 2.5% autologous Platelet Rich Plasma (PRP); a constant volume (20 μl) of the same PRP added culture medium was applied in the central skin loss region, too.

The media in the wells, both the control and the experimental solutions, were changed every two days, while 20 μl of the same medium were added daily into the central injury. Both the control and the treated samples were incubated in an atmosphere of 95% humidified air with 5% CO_2_ at 37°C for 1 (T_1_), 3 (T_3_), 5 (T_5_), and 10 (T_10_) days.

### Assessment Modalities

#### Morphological Analysis

Morphological analysis of the samples was performed by two independent operators at 0 h (T_0_) to identify the histological features of the untreated skin (US) and on days 1, 3, 5, and 10 (T_1_-T_3_-T_5_-T_10_) thereafter to identify the features of the skin in the different experimental conditions. The skin samples were fixed with 10% neutral buffered formalin (pH 7.2) (Bio-Optica, Milan, Italy) for 24 h at room temperature, then they were dehydrated through graded concentrations of ethanol (ASP300S Leica Microsystems Srl, Buccinasco, Milan, Italy) and embedded in paraffin. Subsequently, 3 μm specimens sections were obtained with an automatic microtome (HM355S Thermo Fisher Scientific, Waltham, MA, USA), rehydrated and subsequently processed with the following different specific staining methods.

#### Hematoxylin and Eosin

Hematoxylin and Eosin staining was performed with Harry's Hematoxylin (Bio-Optica) for 4 min and Eosin (Bio-Optica) for 4 min at room temperature (ST5020 and CV5030 Leica Microsystems).

#### Masson's Trichrome Staining

Following rehydration, the sections were treated with 6 drops of Weigert's iron Hematoxylin solution (reagent A) and 6 drops of Weigert's iron Hematoxylin solution (reagent B) for 10 min. After removing the A and B reagents, the sections were treated with 10 drops of picric acid alcoholic solution (reagent C) for 4 min. After a fast washing with distilled water, 10 drops of Ponceau acid Fuchsin according to Mallory (reagent D) were added for 4 min. After a further washing, the sections were treated with 10 drops of Phosphomolybdic acid solution (reagent E) for 10 min and, after removing the latter reagent, 10 drops of Masson Aniline Blue (reagent F) were added for 5 min (all reagents from Bio-Optica). Finally, the sections were washed with distilled water, dehydrated, mounted and observed.

#### Weigert Staining

Following rehydration, the sections were treated with 5 drops of Potassium Permanganate solution (reagent A) and 5 drops of acid activation buffer (reagent B) for 5 min. After washing with distilled water, 10 drops of Oxalic acid solution (reagent C) were added for 5 min and then the sections were placed in a box with Weigert's Resorcin-Fuchsin solution (reagent D) and left overnight at room temperature. Following washing, the sections were treated with 10 drops of acid differentiation buffer (reagent E) for 10 min (all reagents from Bio-Optica). Finally, the sections were washed with distilled water, dehydrated and mounted for observation.

#### Ki-67 Staining

Staining for Ki-67 was performed using the Bench Mark XT ICH/ISH kit (Ventana Medical System, Roche, Arizona, USA). The sections were warmed up to 75°C and then dewaxed by using EZ PREP buffer (Roche). Subsequently, after washing with a reaction buffer for 8, 20, and 36 min, the sections were reacted with fixative for 20 min. Subsequently, the temperature was raised to 95°C and the sections were treated with a UV inhibitor for 30 min, to block endogenous peroxidase activity. The samples were then incubated with 2 μg/ml primary rabbit monoclonal antibody (catalog number 790-4286) for 12 min at 37°C. Subsequently, the sections were washed with distilled water, treated with UltraView Universal DAB, further washed with water for 4 min and eventually stained with Harry's Hematoxylin (Bio-Optica) for 4 min. Finally, the sections were washed again with water, dehydrated and mounted for observation.

All of the stained sections were observed with a Zeiss Axiophot optical microscope (Carl Zeiss AG, Oberkochen, Germany) and photographed by DS-5M Nikon Digital Sight (Nikon Corporation, Tokyo, Japan.

## Results

### Hematoxylin and Eosin Staining

**Figure 2:**
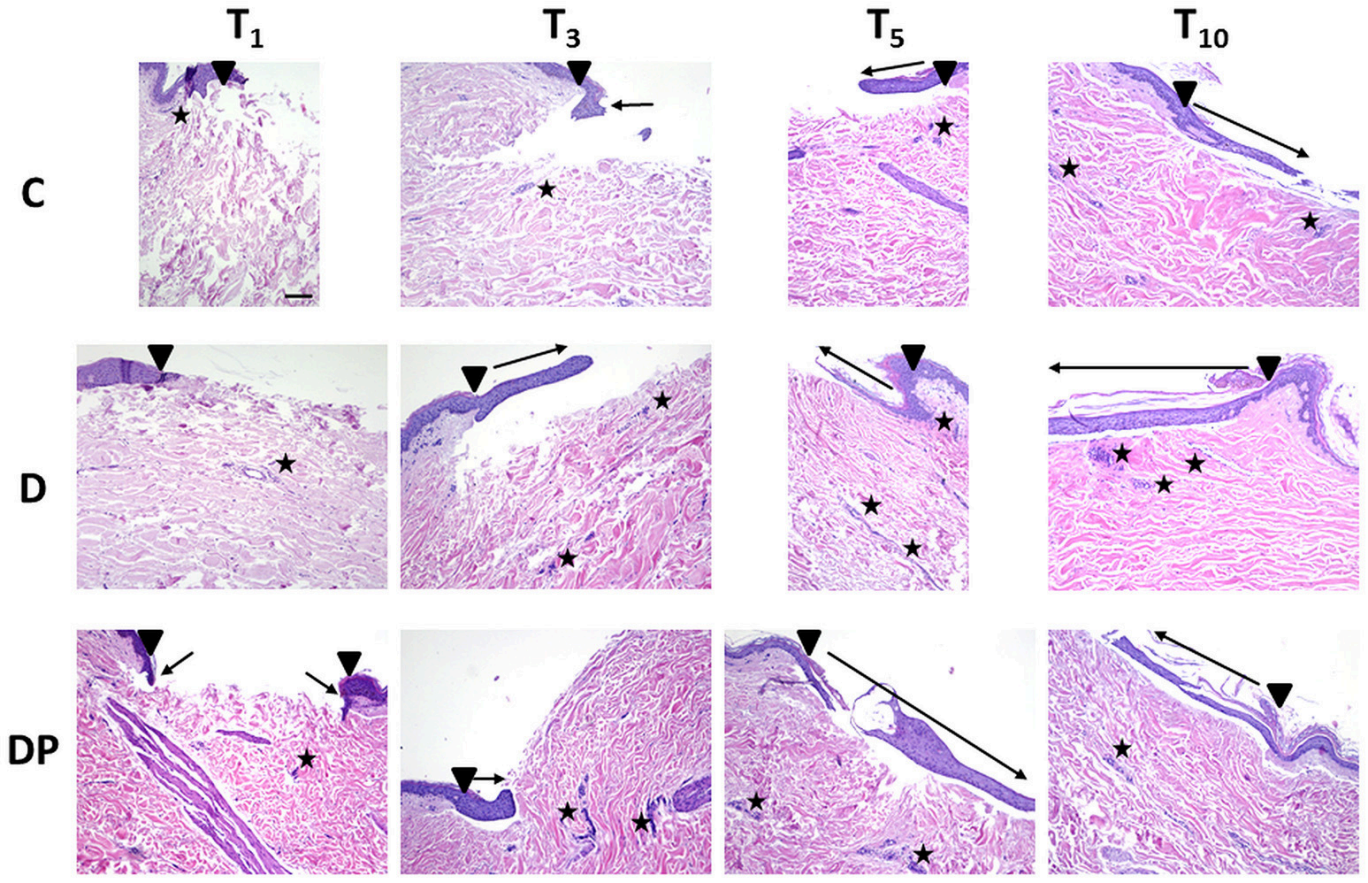
Hematoxylin and Eosin staining in the different culture conditions at the different time points (magnification × 10; bar 100 μm). Black triangle = wound margins; black star = lymph-plasma-cell infiltration; black arrow = re-epithelialization.

The effects of the different culture conditions at different time points with Hematoxylin and Eosin staining are displayed in Figure 2.

#### Control (C)

The regeneration of a multilayered epidermis, including the presence of a stratum corneum and a basal layer pigmentation, was complete at T_10_, but the process was weak and slow up to T_5_.

A few inflammatory plasma cells progressively moved from the perivascular site to the dermis and were still appreciated at T_10_ in both areas.

A few individual fibroblasts were observed up to T_5_ whereas at T_10_ their amount significantly increased with a homogeneous distribution in the dermis, with the exception of the sub-epithelial dermal layer.

A moderate increase of blood vessels was observed at T_5_, particularly around the region of the skin injury.

At T_10_ an evenly distributed rich vascular network was appreciated within the dermis.

The papillary dermis showed an early development from the edge of the lesion toward its center just at T_5_, appearing more structured at T_10_.

At T_10_ the reticular dermis displayed a compact pattern of thick collagen fibers, with chaotic orientation in the superficial layer, while in the deep one, the collagen fibers appeared thinner with an orientation parallel to the skin's surface.

#### DMEM (D)

At T_1_, an early single layered epithelial regeneration was appreciated. Re-epithelialization progressed regularly up to T_10_, when a complete and mature multi-layered regenerated epidermis, including pigmentation of the basal layer and presence of the stratum corneum, was observed.

A few inflammatory plasmacells progressively moved from the perivascular site to the wound, reaching a peak at T_5_.

As in the controls, a moderate increase in the fibroblast count in the reticular dermis was eventually appreciated at T_10_.

A progressive remarkable increase of the vascular network was observed, with an increased concentration in the central wound at T_10_.

At T_3_, early evidence of papillary dermis was observed, progressively developing up to T_10_, when a mature dermal papillary pattern was appreciated. The reticular dermis, loose at T_3_, progressively featured a compact pattern with a blend of thin and well-structured collagen fibers, thicker along the margins of the wound and parallel to the skin's surface.

#### DMEM and PRP (DP)

An initial epithelial advancement was appreciated at T_3_, and at T_5_ a multilayered regenerated epithelium with an early evidence of stratum corneum was appreciated. At T_10_ the regeneration of a multilayered epidermis with evidence of a pigmented basal layer and stratum corneum was complete.

The inflammatory lymph-plasma-cellular infiltration was minimal at all times and mainly located in the perivascular environment, dropping down to a minimal evidence at T_10_.

The fibroblast count was constantly moderate, mainly concentrated in the central portion of the wound.

An evident vascular network was appreciated at T_3_, turning more evident at T_5_ and T_10_, particularly in the region of the skin injury.

At T_3_ an early development of papillary dermis and a compact reticular dermis with a blend of thin and thick collagen fibers were appreciated. The fibers' orientation was perpendicular to the skin surface along the margins of the wound, and parallel in its central portion.

At T_5_ all of the fibers had turned parallel to the skin surface everywhere in the wound.

At T_10_ a completely developed papillary dermis and a compact reticular dermis featuring thick collagen fibers with a parallel orientation to the skin's surface were appreciated.

### Masson's Trichrome Staining

**Figure 3:**
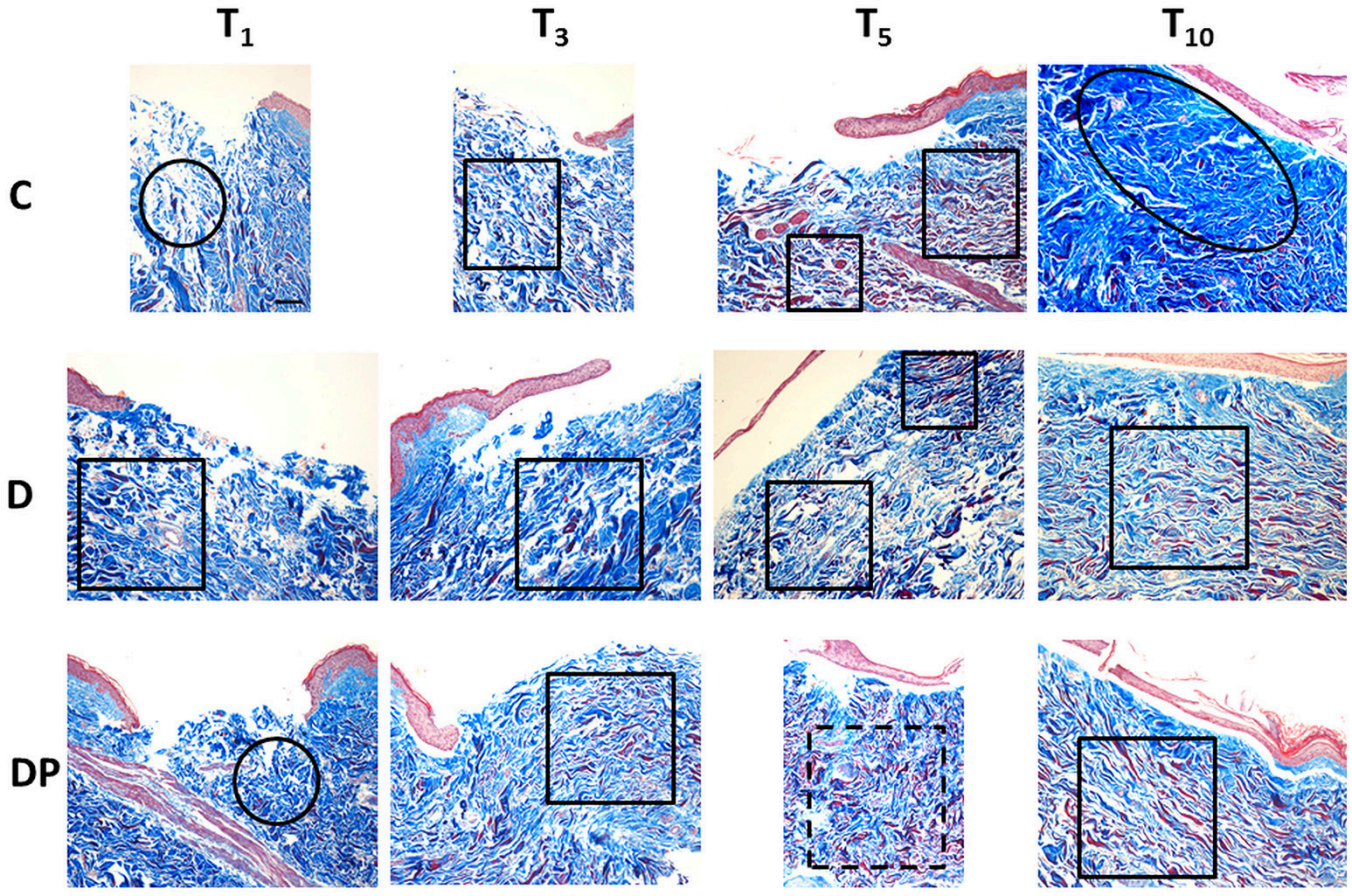
Masson's trichrome staining in the different culture conditions at the different time points (magnification × 10; bar 100 μm). Black circle = chaotic reticular collagen fibers' orientation; black oval = hypertrophic dermis; square = parallel reticular collagen fibers' orientation; dotted line square = perpendicular reticular collagen fibers' orientation.

Figure 3 displays the effects of the different culture conditions at different time points with Masson's trichrome staining.

#### Control (C)

The staining confirmed the first signs of papillary dermis regeneration at T_5_. At the same time, new regenerated fibers were observed also in the reticular dermis. Surprisingly, many of them appeared red stained.

At T_10_ the papillary dermis regeneration had progressed significantly and a marked collagen fiber hypertrophy, with a compact pattern and presence of keloid bodies, was appreciated in the sub-epithelial region of the central wound. Thick and poorly organized collagen fibers were appreciated along the sides of the central wound and thick, red stained, collagen fibers were demonstrated in the deep reticular dermis.

#### DMEM (D)

As in controls, an early papillary dermis was appreciated just at T_5_. At this time, the extracellular matrix displayed an overall decrease, and a blend of thin and thick collagen fibers with an orientation parallel to the skin's surface was appreciated. A marked amount of evenly distributed red stained, thick collagen fibers was appreciated.

At T_10_ a mature and well-structured papillary dermis was observed while a relevant hypertrophy of thick and compact collagen fibers—with chaotic orientation and keloid bodies—was appreciated in the sub-epithelial area of the central wound. Deep in the central wound and along its sides, the collagen fibers appeared thick and still poorly organized. Thick, red stained collagen fibers were appreciated in the deep reticular dermis.

#### DMEM and PRP (DP)

The pattern observed with EE staining confirmed the Masson's staining. At T_3_ an early regeneration of the papillary dermis was appreciated. A blend of thin and thick collagen fibers, with an orientation parallel to the skin's surface, and a small amount of investing extracellular matrix were observed in the reticular dermis. An increased amount of evenly distributed thick, red stained collagen fibers was appreciated in the whole dermis.

At T_5_ the regeneration of the papillary dermis progressed and the reticular dermis displayed an overall compact pattern. A blend of thin and thick collagen fibers with an orientation perpendicular to the skin's surface, and a further reduction of the investing extracellular matrix, were appreciated. A further increased amount of evenly distributed thick, red stained collagen fibers was appreciated in the whole dermis.

At T_10_ the papillary dermis displayed a mature pattern while the reticular dermis was compact. The collagen fibers were thick, with an orientation parallel to the skin's surface and the inter-fibrillar spaces were reduced. A slightly decreased amount of evenly distributed thick, red stained collagen fibers was appreciated in the reticular dermis.

### Weigert Staining

**Figure 4:**
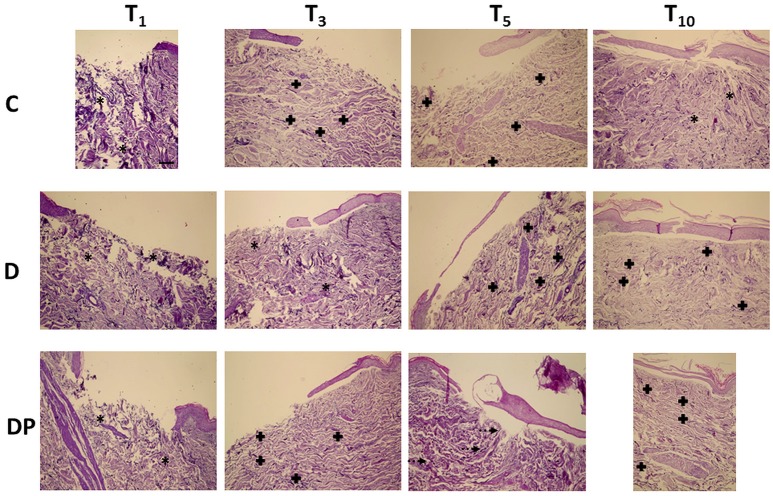
Weigert staining in the different culture conditions at the different time points (magnification x 10; bar 100 μm). Black asterisk = poorly organized elastic fibers; black cross = parallel elastic fibers' orientation; dotted arrow = perpendicular elastic fibers' orientation.

Figure 4 displays the effects of the different culture conditions at different time points with Weigert staining.

#### Control (C)

The overall amount of elastic fibers progressively decreased, up to T_10_, when a minimal amount of unevenly distributed and poorly organized fibers was appreciated.

#### DMEM (D)

As in controls, despite a slight increase of the elastic fibers at T_3_ and T_5_, at T_10_ only a minimal amount of fragmented fibers was appreciated, although a parallel orientation to the skin's surface was maintained.

#### DMEM and PRP (DP)

Although the overall amount of elastic fibers was minimal at any time, at T_10_ they displayed a well-structured pattern, a homogeneous distribution in the whole dermis and an orientation parallel to the skin's surface.

### Ki-67 Staining

**Figure 5:**
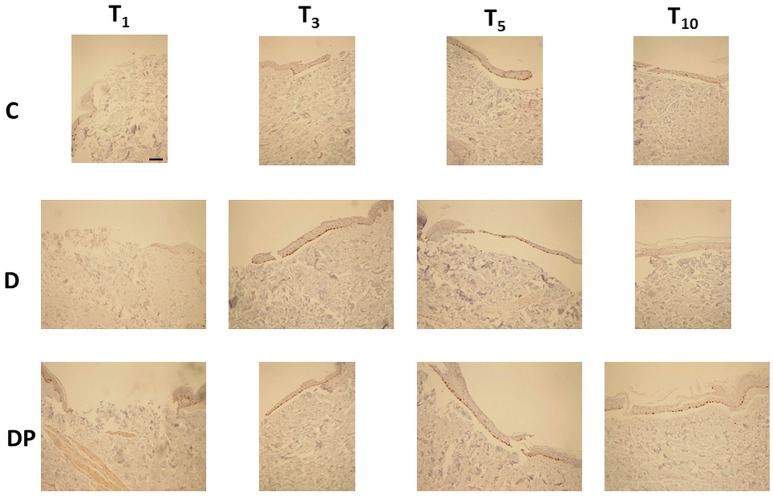
Ki-67 staining in the different culture conditions at the different time points (magnification × 10; bar 100 μm). In brown color the Ki-67-positive nuclei.

The effects of the different culture conditions at different time points with Ki-67 staining are displayed in Figure 5.

#### Control (C)

The regenerative re-epithelialization process was demonstrated by the Ki-67 positive nuclei, observed just along the margins of the wound at T_3_ and progressively distributed in the newly formed tissue filling the central wound up to T_10_.

#### DMEM (D)

Ki-67 positive nuclei were progressively observed from T_1_ up to T_5_. By contrast, at T_10_ they were no longer appreciated.

#### DMEM and PRP (DP)

At T_1_ there was absence of any re-epithelialization and Ki-67 positive nuclei were appreciated just along the margins of the wound. They were early appreciated at T_3_; thereafter they appeared increased at T_5_ but tended to decrease at T_10_. Tables 1 and 2 display the semi-quantitative analysis of the cellular and the connective tissue features, respectively.

**Table 1:** Semi-quantitative analysis of the cellular features.

		**T_**1**_**	**T_**3**_**	**T_**5**_**	**T_**10**_**
C	Epithelial cell advancement front	0	+	++	+++
	Multi-layered re-epithelialization	0	+	+	+++
	Ki-67-positive nuclei	0	+	++	++
	Lymph-plasma-cell infiltration	+	+	+	++
	Fibroblast infiltration	+	+	+	++
	Vascularization	+	+	++	+++
D	Epithelial cell advancement front	+	++	+++	+++
	Multi-layered re-epithelialization	0	++	+	+++
	Ki-67-positive nuclei	+	+++	+++	0
	Lymph-plasma-cell infiltration	+	++	+++	+++
	Fibroblast infiltration	++	++	+	++
	Vascularization	+	++	+++	+++
DP	Epithelial cell advancement front	0	++	+++	+++
	Multi-layered re-epithelialization	0	+	++	+++
	Ki-67-positive nuclei	+	++	+++	++
	Lymph-plasma-cell infiltration	+	++	++	+
	Fibroblast infiltration	++	+	++	+
	Vascularization	+	++	+++	+++

Epithelial cell advancement front: 0 = absent, + = initial, ++ = advanced, +++ = complete;

Multi-layered re-epithelialization: 0 = absent, + = low, ++ = good, +++ = mature;

Ki-67-positive nuclei: 0 = absent, + = present at the margins, ++ = diffuse, +++ = abundant;

Lymph-plasma-cell infiltration: + = minimum, ++ = moderate, +++ = abundant;

Fibroblast infiltration: + = minimum, ++ = moderate, +++ = abundant;

*Vascularization: + = poor, ++ = discrete, +++ = abundant*.

**Table 2:** Semi-quantitative analysis of the connective tissue features.

		**T_**1**_**	**T_**3**_**	**T_**5**_**	**T_**10**_**
**C**	Papillary dermis	0	0	+	++
	Reticular dermis	+	++	+++	++++
	Masson's trichrome blue stained collagen fibers' thickness	+	++	++	++++
	Masson's trichrome red stained collagen fibers' thickness	+++	+++	+++	+++
	Reticular collagen fibers' orientation	+	+++	+++	+
	Elastic fibers' amount	+++	++	+	++
	Elastic fibers' integrity	+++	++	++	++
	Elastic fibers' orientation	+	+++	+++	+
D	Papillary dermis	0	+	++	+++
	Reticular dermis	++	++	++	+++
	Masson's trichrome blue stained collagen fibers' thickness	++	+++	+	++
	Masson's trichrome red stained collagen fibers' thickness	+++	+++	+++	+++
	Reticular collagen fibers' orientation	+++	+++	+++	+++
	Elastic fibers' amount	+++	++	++	+
	Elastic fibers' integrity	++	++	++	+
	Elastic fibers' orientation	+	+	+++	+++
DP	Papillary dermis	0	+	+	++
	Reticular dermis	++	+++	+++	+++
	Masson's trichrome blue stained collagen fibers' thickness	++	++	++	+++
	Masson's trichrome red stained collagen fibers' thickness	+++	+++	+++	+++
	Reticular collagen fibers' orientation	+	+++	++	+++
	Elastic fibers' amount	++	++	++	++
	Elastic fibers' integrity	++	+	++	+++
	Elastic fibers' orientation	+	+++	++	+++

Papillary dermis: 0 = absent, + = initial, ++ = advanced, +++ = mature;

Reticular dermis: + = very loose, ++ = partially loose, +++ = thick, ++++ = hypertrophic;

Masson's trichrome blue and red stained collagen fibers' thickness: + = thin fibers, ++ = blended thin and thick fibers, +++ = thick fibers, ++++ = very thick fibers;

Reticular collagen fibers' orientation: + = chaotic, ++ = perpendicular to the skin surface, +++ = parallel to the skin surface;

Elastic fibers' amount: + = minimal, ++ = moderate, +++ = abundant;

Elastic fibers' integrity: + = fragmented fibers, ++ = blended fragmented and long fibers, +++ = long fibers;

*Elastic fibers' orientation: + = poorly organized, ++ = perpendicular to the skin surface, +++ = parallel to the skin surface*.

## Discussion

Over the last two decades, following extraordinary progress in biomedical research, Regenerative Medicine has emerged as a new branch of Medical Science whose aim is the regeneration of lost or missing structures and functions of the body. The emphasis on regeneration represents the beginning of a true revolution in Medicine, as the concept of healing by scarring is being progressively replaced by the concept of healing by regeneration. Although such an ambitious goal is still far from being achieved, an increasing number of therapeutical applications already allows for the stimulation of tissue regeneration.

Due to the availability of a large amount of Growth Factors, PRP is a current mainstay in Regenerative Medicine and Surgery, with a growing number of clinical regenerative indications (Stuart et al., 1990; Currie et al., 2001; Marx, 2004; Scevola et al., 2010; Cervelli et al., 2011; Burnouf et al., 2013; Kawase, 2015; Gentile et al., 2016). The appropriate concentration of PRP in our study was suggested by the literature reports on its use in *in-vitro* cultures of human fibroblasts (Okuda et al., 2003; Masuki et al., 2016).

Wound healing is the ideal experimental model for investigating the tissue regeneration process, and an increasing number of active substances with regenerative properties is currently under investigation (Faga et al., 2012; Nicoletti et al., 2015). Undoubtedly, the experimental model used in our study required a complex structure and staff organization that included the active interaction between the Plastic Surgery Unit and the Pathological Anatomy Unit at the Istituti Clinici Scientifici Maugeri and the Laboratory for Cell Cultures of the Department of Molecular Medicine at the University of Pavia. The unavailability of such a complex synergic staff and facility network as we had access to, could be a limiting factor for the diffusion of our method in more basic clinical research/experimental organization environments.

A further major limit of the *ex-vivo* model is the unavoidable individual differences within the sample. In our study, such a limit was overcome by establishing restrictive inclusion criteria, allowing a highly homogeneous sample of skin donors. We excluded from the data analysis the skin specimens derived from one female patient with clinically intrinsic wound healing impairment, that matched a peculiar torpidity of her *in-vitro* cultured fibroblasts. Such a peculiar and unusual finding suggested the potential use of fibroblast *in-vitro* culture as a potential screening method for intrinsic cell torpidities that impair the wound healing process, and would otherwise be undetectable with routine tests (Nicoletti et al., 2018).

Our study allowed for the step-by-step investigation of the effects of Platelet Rich Plasma on the wound healing process. PRP enhanced the overall vitality of the skin samples as demonstrated by the macroscopic persistence of a lively pink color, up to, and even beyond 5 days, while the samples, cultured in the other conditions displayed a shift toward a yellow cadaveric color well before this time. The Platelet Rich Plasma constantly stimulated a regular re-epithelialization of a wound and favorably modulated the fibroblast proliferation. Furthermore, with regard to the lymph-plasma-cells, the observed figures suggested a relevant antigenic stimulation induced by the DMEM surplus in comparison with the controls. Such an immune stimulation, seemed to be balanced by the Platelet Rich Plasma in the samples DP, thus suggesting a relevant PRP mediated anti-inflammatory action.

The samples treated with PRP (DP) displayed a regular and compact dermal pattern of prevalent thin collagen fibers in a shorter time vs. the other experimental conditions. Thin and thick collagen fibers constantly co-existed in the samples DP and a shift toward the prevalence of the thick ones was appreciated only at day 10—much later than in the other experimental conditions. Nevertheless, an unexplained peculiar trend in the orientation of the collagen fibers was appreciated: the fibers ran parallel to the skin's surface till day 3, then they orientated perpendicular to the surface at day 5 and came back parallel, as before, at day 10. A possible explanation might be the active interference of Platelet Rich Plasma in the process of de-structuration and re-organization of the collagen fibers. Interestingly, both in the samples D and DP, the dermis did not display signs of scar hypertrophy. The samples DP displayed no decrease in the amount of elastic fibers and a progressive reduction of their fragmentation. The overall orientation of the elastic fibers repeated the pattern of the collagen ones at the same times: it was chaotic at day 1, parallel to the skin's surface at day 3, perpendicular to the surface at day 5 and parallel again at day 10. Although such a configuration is difficult to explain, it might be hypothesized that the Platelet Rich Plasma promotes a more physiologic three-dimensional pattern of the elastic fibers, too. At different times, all of the samples displayed an intense, red staining of some collagen fibers with the Masson's trichrome staining. This intense, red staining might be due to a reduction of the collagen fibers permeability (Bancroft and Stevens, 1982; Wall) and it is unexplained at this time whether it might be related to a maturation or, alternatively, a degeneration process of the fibers themselves.

The *ex-vivo* wounded skin culture protocol developed in this study might be considered a basic experimental model suitable for the assessment of the regenerative properties of different active principles.

## Conclusion

Our experience allowed for the histological and immune-histo-chemical demonstration of the step by step regenerative effects of Platelet Rich Pasma on the human skin wound repair and regeneration process.

The addition of Platelet Rich Plasma to a conventional DMEM culture medium specifically allowed for a favorable modulation of the epithelial cells and fibroblasts proliferation, and a relevant anti-inflammatory action. Platelet Rich Plasma also demonstrated an inhibitory effect on both the collagen and elastic fibers' de-structuration and a favorable modulation of the re-organization of these fibers.

Our research developed a reliable *ex-vivo* skin culture model, with extended vitality, that might permit a more complete assessment of the physiological interaction of topical active principles with the human skin regeneration process.

## Author Contributions

GN contributed to the study design, analyzed the results, and wrote the article. MS carried out the experiments, performed the histological assessments, and contributed to the article writing. LV supervised, coordinated, and performed the histological assessments. AR carried out the experiments. MT was in charge of specimen supply, gathering the data, and contributed to the data analysis and to the writing of the article. AF conceived the study, coordinated the experiments, analyzed the results, and wrote the article.

### Conflict of Interest Statement

LV was employed by Istituti Clinici Scientifici Maugeri, Pavia, Italy. The remaining authors declare that the research was conducted in the absence of any commercial or financial relationships that could be construed as a potential conflict of interest.
